# Rabbit derived VL single-domains as promising scaffolds to generate antibody–drug conjugates

**DOI:** 10.1038/s41598-023-31568-x

**Published:** 2023-03-24

**Authors:** Ana S. André, Joana N. R. Dias, Sandra Aguiar, Sara Nogueira, Pedro Bule, Joana Inês Carvalho, João P. M. António, Marco Cavaco, Vera Neves, Soraia Oliveira, Gonçalo Vicente, Belmira Carrapiço, Berta São Braz, Barbara Rütgen, Lurdes Gano, João D. G. Correia, Miguel Castanho, Joao Goncalves, Pedro M. P. Gois, Solange Gil, Luís Tavares, Frederico Aires-da-Silva

**Affiliations:** 1 CIISA-Centre for Interdisciplinary Research in Animal Health, Faculty of Veterinary Medicine, University of Lisbon, Avenida da Universidade Técnica, 1300-477 Lisbon, Portugal; 2 Associate Laboratory for Animal and Veterinary Sciences (AL4AnimalS), 1300-477 Lisbon, Portugal; 3 Research Institute for Medicines (iMed.ULisboa), Faculdade de Farmácia, Universidade de Lisboa, Lisbon, Portugal; 4 Instituto de Medicina Molecular-João Lobo Antunes, Faculdade de Medicina, Universidade de Lisboa, Avenida Professor Egas Moniz, 1649-028 Lisboa, Portugal; 5 Technophage SA, Avenida Professor Egas Moniz, 1649-028 Lisboa, Portugal; 6 Veterinary Teaching Hospital, Faculty of Veterinary Medicine, Universidade de Lisboa, Av. da Universidade Técnica, 1300-477 Lisboa, Portugal; 7 Department of Pathobiology, Clinical Pathology Unit, University of Veterinary Medicine, Vienna, Austria; 8 Centro de Ciências e Tecnologias Nucleares, Departamento de Engenharia e Ciências Nucleares, IST, Universidade de Lisboa, Estrada Nacional 10, 2695-066 Bobadela LRS, Portugal

**Keywords:** Biotechnology, Cancer, Chemical biology, Drug discovery

## Abstract

Antibody–drug conjugates (ADCs) are among the fastest-growing classes of therapeutics in oncology. Although ADCs are in the spotlight, they still present significant engineering challenges. Therefore, there is an urgent need to develop more stable and effective ADCs. Most rabbit light chains have an extra disulfide bridge, that links the variable and constant domains, between Cys80 and Cys171, which is not found in the human or mouse. Thus, to develop a new generation of ADCs, we explored the potential of rabbit-derived VL-single-domain antibody scaffolds (sdAbs) to selectively conjugate a payload to Cys80. Hence, a rabbit sdAb library directed towards canine non-Hodgkin lymphoma (cNHL) was subjected to in vitro and in vivo phage display. This allowed the identification of several highly specific VL-sdAbs, including C5, which specifically target cNHL cells in vitro and present promising in vivo tumor uptake. C5 was selected for SN-38 site-selective payload conjugation through its exposed free Cys80 to generate a stable and homogenous C5-DAB-SN-38. C5-DAB-SN-38 exhibited potent cytotoxicity activity against cNHL cells while inhibiting DNA-TopoI activity. Overall, our strategy validates a platform to develop a novel class of ADCs that combines the benefits of rabbit VL-sdAb scaffolds and the canine lymphoma model as a powerful framework for clinically translation of novel therapeutics for cancer.

## Introduction

The emergence of monoclonal antibodies (mAbs)-based therapies revolutionized cancer treatment by specifically targeting cancer cells. To date, approximately thirty mAbs have been approved for cancer treatment by the US Food and Drug Administration (FDA), however most mAbs do not possess clinical efficacy as single agents and are currently used in combination with conventional chemotherapy^1–3^. The advances of chemical biology over the last decades allowed progress in a diversity of antitumor molecules (e.g., antibody–drug conjugates, radiopharmaceuticals and immunotoxins)^4^. One of the emerging class of mAb-based targeted therapies are a novel class of anticancer treatment agents called antibody–drug conjugates (ADCs). Broadly, an ADC consists of an antibody attached by a linker to a cytotoxic compound. Due to its components, ADCs combine the targeting, pharmacokinetic and biodistribution properties of antibodies with the cytotoxic potency of small molecules^5^. Nevertheless, the journey towards the development of an effective ADC revealed itself to be long and remarkably challenging.

**Figure 1:**
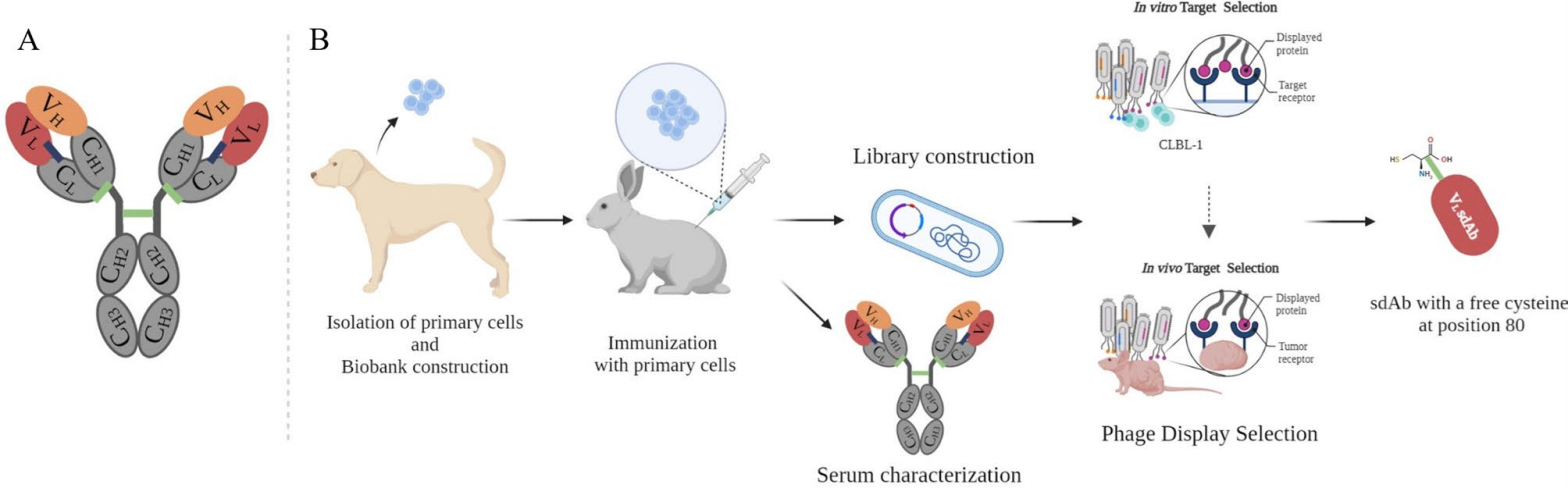
Schematic illustration of the adopted strategy for the development of rabbit derived sdAb ADC. (**A**) Representation of a rabbit IgG antibody. In the present study, we aimed to explore the potential of rabbit-derived VL-sdAbs to develop a new generation of ADCs. Rabbit IgG contains two identical light chains paired with two identical heavy chains. The light chain is composed of an N-terminal variable domain (VL) (red), followed by one constant domain (C_L_). The heavy chain consists of an N-terminal variable domain (V_H_) (orange), followed by three constant domains (C_H1_, C_H2_ and C_H3_). C_H1_ and C_H2_ are linked via a flexible hinge region and contains three disulfide-bridges (green). Most rabbit kappa light chains of the K1 isotype have an unusual disulfide bridge (blue) that joins the variable and constant domains, usually through cysteine residues at positions 80 and 171. This disulfide bridge links framework region 3 of the variable kappa light chain domain with the constant kappa light chain domain, a linkage not seen in mouse or human antibodies. Thus, when the VL-sdAb is isolated the conserved cysteine at position 80 (Cys80, normally paired with Cys171 in an IgG format) becomes exposed at the protein surface and its sulfhydryl group becomes free to be explored to selectively conjugate a chemical payload, without requiring further genetic engineering manipulation. (**B**) Representation of the antibody selection process. Aiming to develop a new ADC molecule, we firstly immunized one female New Zealand white rabbit with lymph node primary cells derived from a canine multicentric lymphoma biobank previously established^34^. To induce a strong immune response, the rabbit was immunized and boosted with 1 × 10^7^ of cNHL of primary cells isolated from the lymph node of ID5 and ID6 patients diagnosed with Diffuse Large B-Cell Lymphoma (DLBCL). Tumor cells isolated from the patients were thawed, washed and resuspended in PBS. Lymph nodes cNHL primary cells were administered subcutaneously at 2–3 weeks for 3 months. Following the rabbit immunization and validation of the immune response, an immune VL-sdAb library was constructed. To select the best antibodies for NHL targeting, sdAb library was used for an in vitro whole cell and in vivo phage display in a xenograft CLBL-1 murine model. After, the phage display, NGS and sanger sequencing analysis a panel of VL-sdAbs were selected. The three most promising VL-sdAbs were characterized by flow cytometry and immunofluorescence microscopy and then the most promising VL sdAb was chosen to be conjugated to the SN-38 using the free exposed Cys80.

The main challenges in ADC development, including those already on the market, consist of engineering issues that vary from design to production. These problems often lead to heterogeneous products containing a mixture of species with different drug-to-antibody ratios (DARs)^6,7^. This heterogeneity results in variable pharmacokinetic and therapeutic profiles, leading to CMCs (Chemistry, Manufacturing and Controls) challenges^6,8,9^. This issue is mostly associated with conventional drug bioconjugation methods that rely on multiple lysine modifications, or on the functionalization of thiols generated by the reduction of interchain disulfide bonds^6,8,9^. Conventional mAbs display more than 80 lysine residues, but only 30–40 are accessible to solvent modifications and only 8 cysteines can be modified in a conventional IgG. Moreover, conserved cysteines play a fundamental role in the antibody structure and its use in conjugation often leads to aggregation issues and misfolding^6,8–11^. New approaches have been used to overcome these drawbacks, including site-specific conjugation methods, which have resulted in a new generation of more uniform ADCs. Yet, most of these methodologies are not compatible with the scale-up of the manufacturing process required for ADC production. Therefore, further improvements to ADC design and development are required to allow the synthesis of more homogeneous and stable molecules with higher therapeutic indexes^12–16^. Most ADCs currently in development and on the market consist of a complete IgG antibody. However, the clinical use of these IgG-based products has been hampered by the low penetration in tumor tissues due to their high molecular weight, and by the high manufacturing costs in mammalian cells^6,17^. Moreover, there is now evidence that the Fc domain of an IgG may be redundant or even unfavorable for ADCs efficacy^17^. In fact, ADCs prolonged half-life promoted by the neonatal Fc receptor (FcRn) increases exposure to healthy tissues, while FcγR cross-reacts with endothelial and immune cells, both of which are biological processes related to off-target toxicity^6^. A promising alternative to the conventional immunoglobulin (IgG) to produce ADCs are smaller formats, such as single-domain antibodies (sdAbs), single-chain antibody fragments (scFvs) and minibodies^18,19^. sdAbs are presently the smallest functional antigen-binding fragments, only consisting of a VH or VL, that can be obtained from conventional IgGs. These small-size scaffolds of about 15 kDa present higher tumor penetration and accessibility to targets not easily reached by large-size conventional mAbs^20–22^. Furthermore, their faster clearance rate compared to the intact IgG, may be advantageous in cases where the risk of toxicity in healthy tissues increases with prolonged exposure^9^. In addition to their reduced size, sdAbs also present higher stability and solubility, while decreasing the number of potentially immunogenic epitopes^23^. Over the past years, we have been showing the great potential of rabbit-derived sdAbs for several therapeutic applications^24–30^. Rabbit-derived sdAbs have all the promising properties of smaller antibody fragments and, in addition, have a unique characteristic in their light chain variable domains (VL) that make them promising scaffolds to develop ADCs (Fig. 1A).

Most rabbit kappa light chains have an unusual disulfide bridge that joins the variable and constant domains, usually through cysteine residues at positions 80 and 171 (Fig. 1A). This disulfide bridge links framework region 3 of the variable kappa light chain domain with the constant kappa light chain domain, a linkage not seen in mouse or human antibodies. Therefore, when the VL sdAb is isolated the conserved cysteine at position 80 (Cys80, normally paired with Cys171 in an IgG format) becomes exposed at the protein surface and its sulfhydryl group becomes free to be explored to selectively conjugate a chemical payload, without requiring further genetic engineering manipulation^29,31–33^. Furthermore, rabbit derived VL sdAbs present promising advantages in terms of manufacturing costs and downstream process since they can be expressed in bacterial systems and purified by protein L. Within this context, the present study aimed to explore the properties of rabbit-derived VL sdAbs to develop a new generation of ADCs for cancer treatment. For this purpose, we developed a VL sdAb ADC with a potent cytotoxic payload, SN-38, that was conjugated to the free Cys80. As illustrated in Fig. 1B, to validate our strategy, we developed an ADC against canine non-Hodgkin lymphoma (cNHL), an animal model recently proposed as a powerful framework for clinically relevant translation of novel therapeutic molecules and approaches.

## Results

### Rabbit immunization, antibody library construction and phage display selection

**Figure 2:**
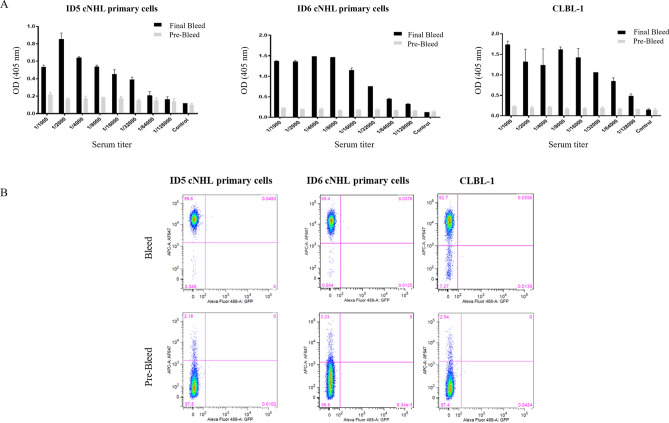
Characterization of rabbit immune response. To evaluate the rabbit immune response, antibody titers were monitored by a cell ELISA and flow cytometry analysis using the cNHL primary cells and CLBL-1 cell line. Pre-bleed sera was used as control. (**A**) Serum titration by ELISA. 5 × 10^4^ cells were incubated with serial dilutions of rabbit serum (from 1/1000 to 1/32,000). Cells were incubated with secondary antibody goat-α anti-rabbit IgG-Fc specific HRP at 1/3000. The rabbit presented a high response against cNHL primary cells and CLBL-1 cell line with a high serum titer (1/64,000), in opposition to the pre-bleed serum. (**B**) Binding properties against cNHL cells were also analyzed by flow cytometry. Cells were incubated with the rabbit pre-bleed and final bleed (1/3000) and with the secondary antibody Alexa Fluor 647 Goat Anti-Rabbit IgG (1/10,000). The flow cytometry analysis confirmed a high serum activity of the rabbit against the primary cNHL cells (ID5 and ID6 patient cells) and CLBL-1 cell line.

Aiming to develop a highly specific rabbit-derived V_L_ sdAb against cNHL, a whole-cell rabbit immunization strategy, antibody library construction, and phage display selection were performed, as depicted in Fig. 1B. To induce a strong and specific immune response against cNHL receptors, one female New Zealand rabbit was immunized with a pool of two isolated cNHL primary cells (ID5 and ID6) from a canine multicentric lymphoma biobank previously established and characterized by our group^34^. The immunization response, antibody titers and specificity were monitored by cell ELISA and flow cytometry. As shown in Fig. 2A, the results showed that the rabbit presented a final bleed serum with a high specificity against both cNHL primary cells (ID5 and ID6) and a canine B-cell lymphoma stable cell line (CLBL-1)^35,36^. Furthermore, these data demonstrated that immunizations resulted in a strong immune response with a high serum titer (1/64,000), contrarily to the pre-bleed serum. The flow cytometry analysis confirmed the results obtained by ELISA (Fig. 2B).

**Figure 3:**
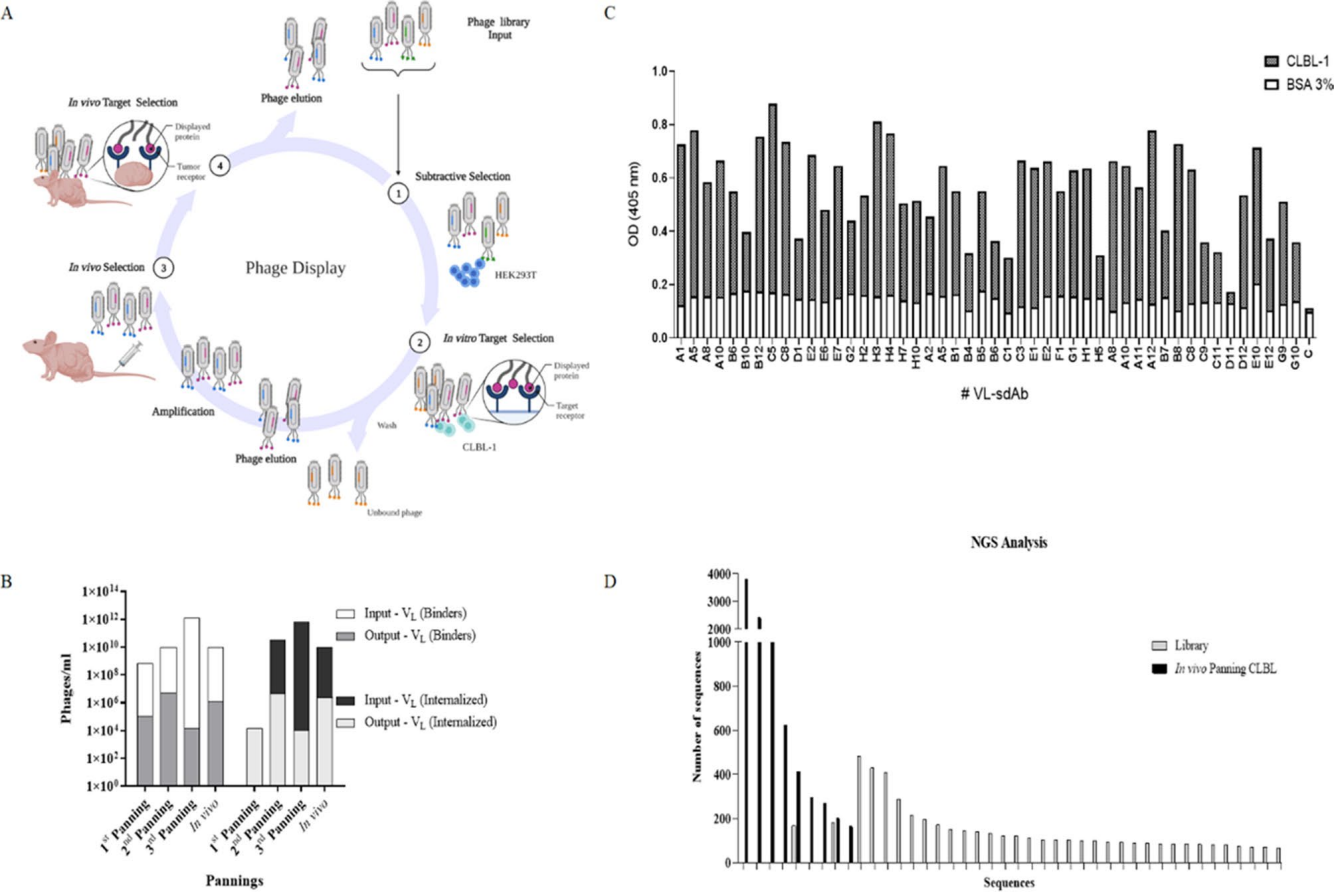
Phage display selection and screening for sdAb for cNHL targeting. (**A**,**B**) To select the best antibodies for NHL targeting, the constructed sdAb immune library with a diversity of 3.4 × 10^8^ was used for an in vitro whole cell and in vivo phage display in a xenograft CLBL-1 murine model. (**A**) Phage library was first panned using a subtractive cell phage display protocol on HEK293T cells followed by a positive selection on CLBL-1 cells. Three rounds of in vitro selections were performed, and an additional panning was performed in vivo in a xenograft CLBL-1 murine model. Briefly, the output phages from the 3rd in vitro panning were recovered, reamplified and tailed vein injected in a xenograft murine cNHL model. After 60 min, the mice were euthanized, and the phages recovered from the tumor. Two different elution methods were performed to recover binder and internalized antibodies. (**B**) The three in vitro pannings resulted in a lower number of phages in the output titers compared to the input titers (~ 10^11–12^ input pfu to ~ 10^3–4^ output pfu). At the end, the in vivo panning presented a titer of 1.2 × 10^6^ of VL sdAbs binders and 2.3 × 10^4^ of VL sdAbs internalizers (phages/mL), indicating a high enrichment towards the phages to target cNHL. (**C**) To select the most promising VL sdAbs, individual clones were autoinduced and the supernatant tested in ELISA assays against CLBL-1 extracts and 3% BSA. The clones that revealed a stronger signal against CLBL-1 were selected. (**D**) To characterize in more detail the enriched sequences during the in vivo phage display, next generation sequencing was performed. Two samples were sequenced: biopanning from CLBL-1 tumor models and initial immune library. Upon bioinformatic analysis, we identified the diversity of each sample and the most represented clones. The number of occurrences was higher in biopannings compared with the initial library, supporting the specificity of the phage display selection that diminishes the high diversity of clones present in the library.

Following the rabbit cell-immunization and validation of the immune response towards cNHL cells, our major goal was to select the most promising antibodies regarding cellular internalization and tumor uptake properties. For that purpose, an immune VL sdAb library was constructed as described in the methods section, originating a phage displayed library with a diversity of 3.4 × 10^8^. Selection of highly specific VL sdAbs with suitable properties for the development of ADCs was then performed using an in vitro and in vivo phage display screening (Fig. 3A). Firstly, a subtractive in vitro cell phage display was performed using the HEK293T cell line, which does not express the cNHL antigens. This cell phage display screening was based on previous Carlos Barbas’^37,38^ and our own studies^25^ and consisted in a whole-cell selection protocol with negative and positive selection steps (subtractive phage display), implemented to remove antibodies reacting with common antigens. The CLBL-1 cell line was used for the positive selection due to its stable expression of cNHL antigens. Three pannings were performed and over the course of selection, stringency was incremented by increasing the number of washes in order to collect the VL-phages with greater target affinity and specificity. Two types of elution methods were used to select binders and internalized antibodies as described in the methods section. As shown in Fig. 3B, and as expected, the three in vitro phage display pannings resulted in a lower number of VL phages in the output titers compared to the input titers (~ 10^11–12^ input to ~ 10^3–4^ output pfu). Furthermore, the biopannings profile indicated that the in vitro phage display successfully led to the enrichment for highly specific cNHL VL sdAbs with binding and cellular internalization properties. Following the in vitro phage display selection, a final in vivo phage display panning was performed in a murine xenograft cNHL model (Fig. 3A). The output phages recovered from the 3rd in vitro panning was recovered, reamplified and tailed vein injected in a xenograft CLBL-1 murine model as previously described^39^. The phages were recovered to retrieve sdAbs that specifically targeted and were uptaken by the grafted cNHL tumors. By allowing the enriched antibody panel to circulate in the mouse and collecting VL phages that targeted the xenograft tumor, we expected to select antibodies that met the defined biological effect, confirming the in vivo availability of the epitopes and tumor targeting. At the end, the phages recovered presented a titer of 1.2 × 10^6^ of VL sdAb binders and 2.3 × 10^4^ of VL sdAb internalizers (phages/mL), which reflected a high enrichment towards phages that effectively target cNHL.

### Screening for VL sdAb towards cNHL targeting

After the phage display selection, to express and select the best anti-cNHL VL sdAbs, phagemid DNA derived from the in vivo output selection was cloned and transformed as described in the methods section. Then, individual clones were auto-induced, and the supernatant tested in ELISA assays against CLBL-1 and Jurkat cell extracts. To select the best lead candidates, three parameters were evaluated: binding against cNHL, expression yields and unspecific binding. Several clones were screened and the VL sdAbs that revealed a stronger signal against CLBL-1 cells were selected (Fig. 3C). After selection, to characterize the selected clones, the eighteen best lead candidates were analyzed by sanger sequencing. The sequences were analyzed as described in the methods section. After the alignment of the eighteen sequences, we concluded that some of the clone sequences were identical, leading to six different clones (data not shown). To get further insights of the enriched sequences during the in vivo phage display selection, we performed next generation sequencing (NGS) of the biopanning repertoires. NGS allows massive sequence analysis of the panning population, enabling a genomic assessment of the library diversity and frequency of each clone. Two samples were sequenced: biopanning from the CLBL-1 tumor models and initial immune VL sdAb cNHL library (Fig. 3D). Upon bioinformatic analysis of the NGS data, we were able to determine the diversity of each sample and identify the most represented clones. A total of 34,880 and 48,864 sequences were obtained for the biopanning from the CLBL-1 tumors and cNHL library, respectively. The number of singletons, consisting of the sequences with a single occurrence, varied between samples, ranging from 76.8% in the library, and 33.3% in the in vivo biopanning from the CLBL-1. Through the comparison of the sequence prevalence between the biopannings and the library, we were able to verify that the number of occurrences in each sequence was higher in the biopanning clones. These results evidenced the specificity of the phage display selection, which resulted in the enrichment of sdAb clones among the highly diverse library. The comparison of ELISA data, sanger sequencing and NGS analysis allowed to select the three best clones (A12, B12 and C5) that were produced and purified. Due to its binding characteristics and production properties (protein yields of 8–10 mg/L), C5 was chosen to be thoroughly characterized and used in the development of the ADC.

### Binding and internalization characterization of C5 VL sdAb

**Figure 4:**
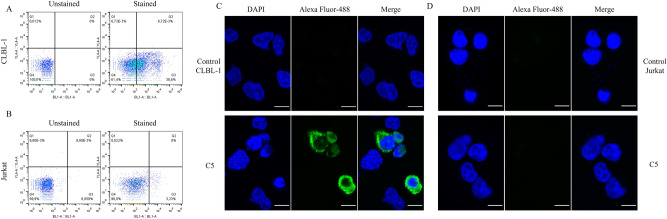
Binding and internalization characterization of C5 VL sdAb. C5 binding and internalization properties against CLBL-1 and Jurkat cells were evaluated by flow cytometry and immunofluorescence. (**A**) For flow cytometry analysis, 1 × 10^6^ of CLBL-1 cells were incubated with Live/Dead reagents for 30 min. Then, 3 µM of C5 were incubated with the cells for 90 min at 37 °C. After, cells were washed, fixed with PFA, permeabilized and incubated for 30 min with anti-HA antibody (1/50), washed twice and incubated with anti-rat Alexa Fluor-488 (1/250). C5 has demonstrated to bind to the CLBL-1 cells. (**B**) 1 × 10^6^ Jurkat cells were exposed to the same protocol as CLBL-1 cells. By contrast, no binding of C5 was detected with Jurkat cells. (**C**) To confirm the binding of C5 to the CLBL-1 verified by flow cytometry, we evaluated its distribution on the cells by immunofluorescence assay. 1 × 10^6^ of CLBL-1 and Jurkat cells were plated on ibidi µ-Slide 8 Well Glass Bottom and incubated with 3 µM of C5 for 90 min. After incubation, cells were washed, fixed, permeabilized, blocked and incubated overnight with rat anti-HA (1/50). Next day, cells were washed and incubated with anti-rat Alexa Fluor-488 (1/500). At the end, DAPI Vectashield was added to the cells. It is possible to observe a high density of C5 labeled with Alexa Fluor-488 in the perinuclear region. (**D**) In contrast, there is no detectable fluorescence in the control image, nor in the Jurkat cells. Representative microphotographs with C5 (green) and DAPI stained-nuclei (blue) are shown.

The C5 binding and cellular internalization properties towards CLBL-1 cells were studied by flow cytometry and immunofluorescence. For the flow cytometry, C5 was incubated with CLBL-1 cells as described in the methods section. As demonstrated in Figs. 4A, C5 VL sdAb, specifically binds to the CLBL-1 cells. On the contrary, no binding interaction of C5 with Jurkat cells, a leukemic T-cell line, was observed (Fig. 4B). Live/dead reagent was used to exclude dead cells and the background noise was also evaluated in the control with the secondary antibody (data not shown). To better characterize the binding of the C5 to the CLBL-1 cells, we further evaluated C5 cellular internalization properties using an immunofluorescence assay. As shown in Fig. 4C, a high density of Alexa Fluor-488 labeled C5 was observed in the perinuclear region. Conversely, there was no detectable fluorescence in the control sample image nor in Jurkat cells (Fig. 4D). Thus, these data confirmed the C5 binding to the CLBL-1 cells and its internalization into the cytoplasm. This cellular internalization feature is essential to develop an efficacious ADC.

**Figure 5:**
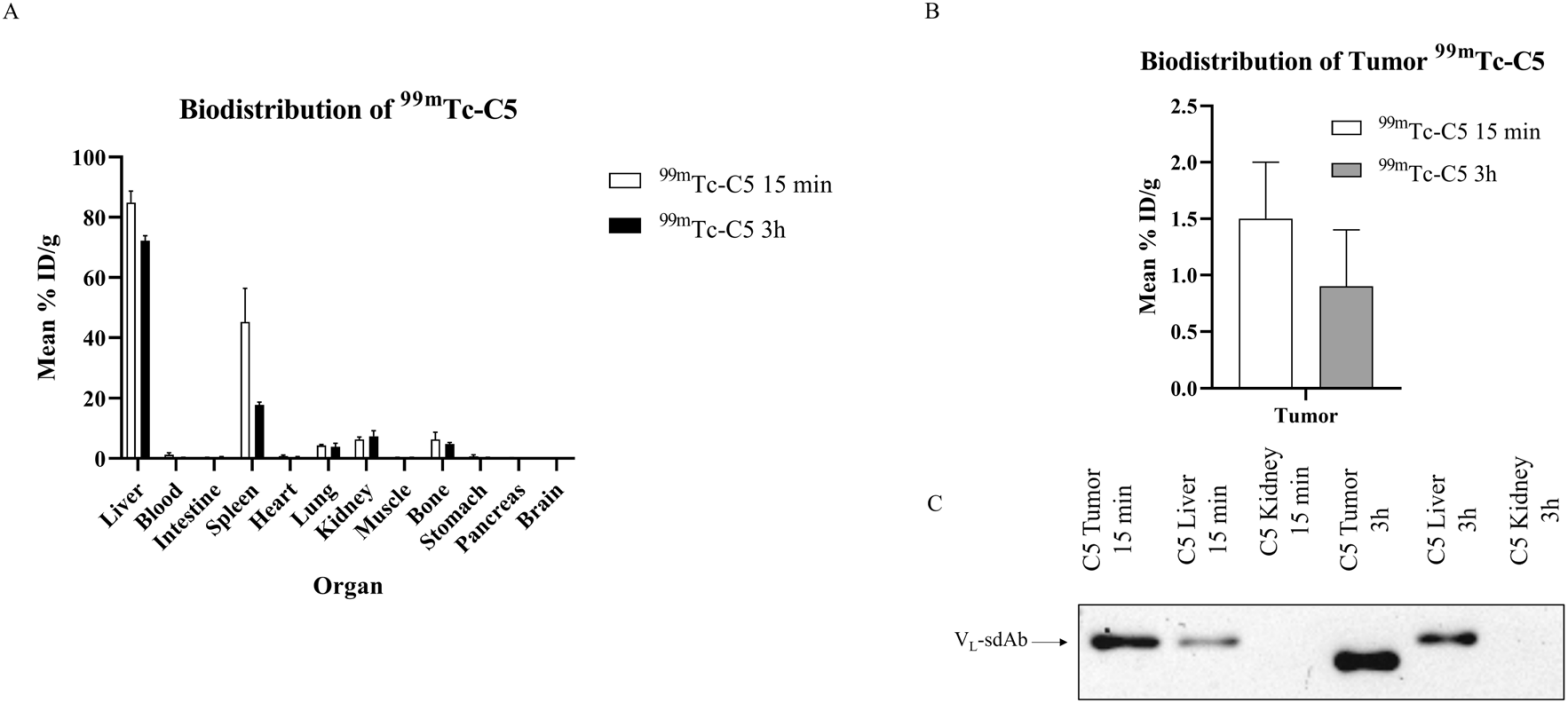
Biodistribution profile of ^99m^Tc-C5. To evaluate the tumoral uptake and pharmacokinetic profile of the C5 VL sdAb, a biodistribution assay was performed on a xenograft model of cNHL at two different time points (15 min and 3 h). C5 was radiolabeled with ^99m^Tc(CO)_3_(H_2_O)_3_ and intravenously injected in the tail vein of a xenograft mouse model of cNHL. Mice were sacrificed at 15 min and 3 h, and the radioactivity of each organ was measured. The activity in each organ was calculated and expressed as a percentage of injected radioactivity dose per gram of organ or tissue (%ID/g). (**A**,**B**) The results show that the tumoral uptake was around 1.5 ± 0.5%ID/g at 15 min, diminishing to 1% at 3 h after injection. A fast elimination in the major organs was noticed, with exception of the liver and spleen. (**C**) The results were also confirmed by western blot analysis. C5 was recovered from the mice organs by immunoprecipitation with His beads and analysed by western blot. Samples were loaded in a 15% SDS page acrylamide gel. Following gel transfer and blocking, the membrane was incubated with HRP-conjugated anti-His antibody (1/3000). Protein detection was performed by chemiluminescence using Luminata ForteWestern HRP and acquired using the ChemiDoc XRS + imaging system (Bio-Rad). This analysis confirmed the presence of the C5 VL sdAb in CLBL-1 xenograft tumors at 15 min and 3 h. Representative blots are shown. Image is part of the original blot, which is depicted in Supplementary Fig. 1.

### Biodistribution studies of C5 VL sdAb

To evaluate the tumor uptake and pharmacokinetic profile of the selected C5 VL sdAb, a biodistribution assay was conducted as described in the methods section. The obtained biodistribution profile of the labeled 99mTc-C5, expressed as %ID/g, is presented in Fig. 5A,B, showing that tumor uptake was around 1.5% ID/g at 15 min, decreasing to 1% at 3 h after injection. In addition, apart from the liver and spleen, the biodistribution data revealed a rapid elimination from blood and major organs, with low levels of activity. These results were also confirmed by western blot analysis that established the presence of the C5 in the CLBL-1 xenograft tumors (Fig. 5C and Supplementary Fig. 1). Thus, the obtained data demonstrated that C5 VL sdAb presented a favorable tumor uptake and biodistribution profile, making it a promising candidate to develop our ADC.

### Development of C5-DAB-SN-38 and evaluation of its activity on cNHL cells

**Figure 6:**
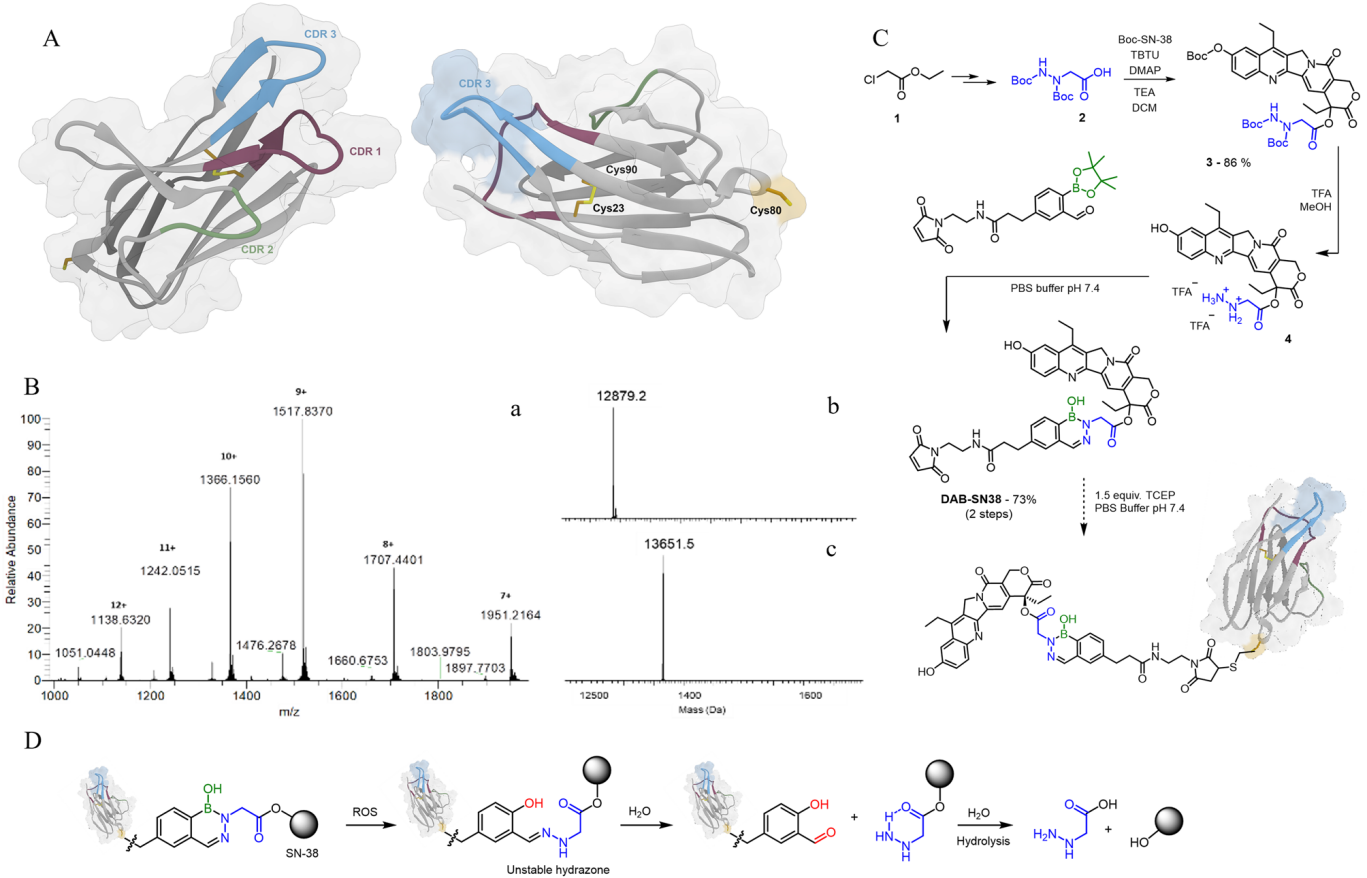
Development of C5-DAB-SN-38 ADC. (**A**) Ribbon representation of C5 sdAb’s predicted 3D structure. The complementarity-determining regions (CDRs) domains have been highlighted in purple (CDR1), green (CDR2) and blue (CDR3). The side chain atoms are shown in stick representation and colored in yellow. The van der Waals surface is depicted in transparent coloring. The surfaces of CDR3 and Cys80 have been highlighted with blue and yellow coloring, respectively. CDR and amino acid numbering were done according to Kabat et al.^54^ (**B**) High-resolution mass spectra of conjugate C5-DAB-SN-38. To a PBS pH 7.4 solution containing C5 (10 µM) and TCEP (1.5 equiv., 3.5 mM), DAB (20 equiv., 9 mM, DMSO) was added and the solution was mixed during 1.5 h at 25 $^\circ$C. The expected conjugate was evaluated after 1.5 h by High-Resolution Mass Spectrometry, recorded in a Thermo Scientific Q Exactive hybrid quadrupole-Orbitrap mass spectrometer (Thermo Scientific Q Exactive Plus). The final immunoconjugate VL-DAB-SN-38 was detected. The mass spectra were deconvoluted using MagTran software. b) Deconvoluted high-resolution mass spectra of C5 (12,879.2 Da). c) Deconvoluted high-resolution mass spectra of conjugate C5-DAB-SN-38 (13,651.5 Da). (**C**) Bioconjugation of C5-DAB-SN-38. Conjugate C5-DAB-SN-38 obtained from C5 cysteine modification at PBS buffer at pH 7.4 (10 µM) at room temperature. (**D**) Oxidation and self-immolative mechanism of ROS-responsive diazaborine linker.

**Figure 7:**
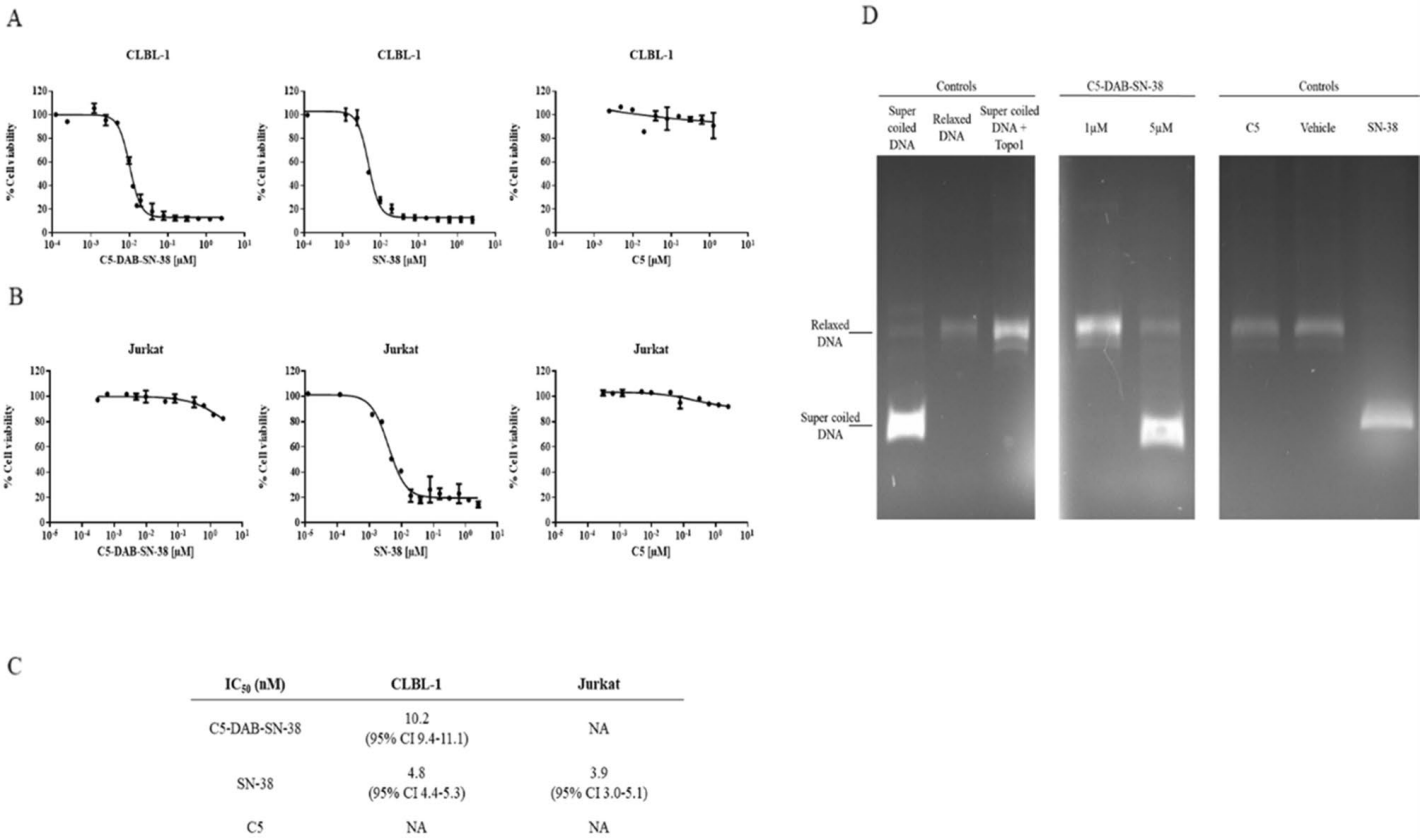
Cytotoxic effects of C5-DAB-SN-38. To determine the effect of C5-DAB-SN-38 on CLBL-1 and Jurkat cells, a cell viability assay was performed using the WST-1 reagent. 6 × 10^4^ cells were seeded and treated with increasing amounts (2.5 μM to 12.5 nM) of each compound (C5, C5-DAB-SN-38 and SN-38). After 48 h treatment, cell viability was assessed. (**A**) C5-DAB-SN-38 demonstrated a dose-dependent cytotoxicity effect on cNHL cells. (**B**) On the other hand, C5-DAB-SN-38 had no effect on Jurkat cells, proving the specificity of the ADC. C5 was used as control. (**C**) Best-fit EC50 values of each formulation were calculated using GraphPad Prism software (version 9.2.0, San Diego, CA, USA) using the log (inhibitor) vs response (variable slope) function.** D**) Effects of C5-DAB-SN-38 on DNA TopoI activity. To evaluate the effects of C5-DAB-SN-38 on DNA Topo I, we evaluated its activity using the Human Topoisomerase I Assay Kit. After incubation of C5-DAB-SN-38 with 1 × reaction buffer and 10 U of Topo I for 1 h at 37 °C, the supercoiled DNA was added. To stop the reaction, stop loading buffer was added. DNA TopoI activity was visualized by 1% agarose gel electrophoresis attained with ethidium bromide. Relaxed DNA indicated TopoI activity, while supercoiled DNA indicated an inhibition. The presence of C5-DAB-SN-38 at higher concentrations inhibits DNA TopoI in a dose dependent manner, empathizing the effect of SN-38, present on the ADC, as a cause of cell death. Relaxed DNA and supercoiled DNA were used as positive/negative controls. C5 and SN-38 were also used as controls. Image is part of the original gel that is presented in Supplementary Fig. 3.

Aiming to develop our ADC by exploring free cysteine residues, we started by modeling the tridimensional structure of C5 VL sdAb using a protein structure prediction software. The obtained structural model revealed a traditional immunoglobulin domain fold composed of eight antiparallel β-strands arranged into two β-sheets, which are connected by a single disulfide bond formed between Cys23 and Cys90, forming a β-sandwich (Fig. 6A). The three complementarity-determining regions (CDRs) motifs are easily identifiable, with CDR3 having the largest area of exposed surface. Importantly, the structure shows the presence of a third cysteine (Cys80) on its surface with a free sulfhydryl group, which is normally involved in a rabbit-unique interdomain disulfide bond with another cysteine on the CL domain^29,31–33^. As predicted, by isolating the VL domain, Cys80 becomes exposed at the protein surface and its sulfhydryl group becomes free to be used in cysteine-based conjugation strategies. As such, C5 single free cysteine was modified with DAB-SN-38, a molecule containing the cytotoxic drug SN-38 and a maleimide group, connected by a ROS-responsive diazaborine linker (Fig. 6B–D). C5 was successfully converted in the homogenous targeting drug conjugate, C5-DAB-SN-38. The expected DAR of 1 was confirmed by reverse phase-HPLC and displayed > 95% of single modified sdAb (Supplementary Fig. 2). The in vitro cytotoxicity of C5-DAB-SN-38 was evaluated on cNHL cells and on irrelevant cells. For that, a cell viability assay on CLBL-1 and Jurkat cells was conducted. C5-DAB-SN-38 exhibited a dose-dependent cytotoxicity on cNHL cells proliferation (Fig. 7A). Importantly, the EC50 value of C5-DAB-SN-38 (10.2 nM; 95% CI 9.4–11.1) was similar to that of SN-38 (4.7 nM; 95% CI 4.2–5.2), showing that conjugation does not interfere with the cytotoxicity of SN-38 (Fig. 7C). By contrast, C5-DAB-SN-38 had no effect in the proliferation of Jurkat cells, a leukemic T-cell line, which demonstrates the ability of C5-DAB-SN-38 to recognize selectively the target cells (Fig. 7B). In addition, the C5 alone does not affect cell proliferation in both cell lines (Fig. 7A,B). The primary mechanism of cell death induced by SN-38 is the inhibition of DNA-Topo I activity, preventing the conversion of supercoiled DNA into a relaxed DNA form. Thus, to confirm if the underlying mechanism of action responsible for the cytotoxic effects of C5-DAB-SN-38 on cNHL cells is maintained, we evaluated its activity on DNA-Topo I. As shown in Fig. 7D (and Supplementary Fig. 3), C5-DAB-SN-38 inhibits DNA-Topo I as evidenced by the presence of the supercoiled DNA form. In contrast, neither C5 nor the vehicle were able to alter the enzyme activity, suggesting that C5-DAB-SN-38 effects and its mechanism of action are related to SN-38 cytotoxicity.

## Discussion

Following decades of research and troubleshooting, several technological advances and a better understanding of the mechanisms underlying ADC activity have resulted in the development of multiple agents that provide significant therapeutic benefit to cancer patients. While advances in this area have been encouraging, the clinical translation of these innovative therapeutics has been hindered by several hurdles related to the complexity of their clinical development. The approval of eight new ADCs over the last five years provided a renewed interest in the potential of this class of drugs. However, the overall low approval rate continues to highlight the need for novel strategies to maximize the potential benefit that ADCs can provide to cancer patients^40^. Thus, several additional therapeutic candidates and new approaches on ADC development are being actively investigated, some of which have the potential to change cancer therapy strategies. One of the most promising approaches to improve the efficiency of ADC is exploring alternative antibody formats for targeted payload delivery. Over the past few years, we have shown the great potential of rabbit-derived sdAbs for several therapeutic applications, as an alternative to conventional mAbs^24–30^. Due to their reduced size and low complexity, as well as the lack of the Fc region, sdAbs have been able to achieve improved tumor penetration and in vivo tolerance compared to traditional ADCs^10,20,24^. Importantly, rabbit VL domains possess a unique intrachain disulfide bridge between cysteine 80 in VL and cysteine 171 in CL, that is not found in human or mouse VL domains. Thus, an isolated VL sdAb presents a free exposed cysteine at position 80 that can be used for payload conjugation without any modification^29,31–33^. This unique characteristic makes rabbit VL sdAbs promising scaffolds to integrate a homogeneous and stable ADC, overcoming the heterogeneity problems associated with conventional drug bioconjugation methods, while surpassing manufacturing costs associated with novel site-specific conjugation methods. Within this context, in the present study we aimed to explore the properties of rabbit-derived VL sdAbs to develop a new generation of highly selective and potent ADC for cancer treatment. To validate our antibody platform, we used the canine lymphoma as an animal model of human NHL. Owing to remarkable similarities with its human counterpart, the canine lymphoma model has been proposed as a powerful framework for rapid and clinically relevant translation of novel immunotherapies. Naturally occurring NHL in dogs present many clinical, pathological, immunologic, molecular, diagnostic and therapeutic similarities to those observed in humans, that are difficult to reproduce in conventional preclinical models. This allows studying the complex immune interactions during the course of treatment while also addressing long-term efficacy and toxicity of cancer immunotherapies^41^. Furthermore, the use of dogs as a model in cancer drug discovery can be mutually beneficial for all parties, since the therapeutics for cNHL are rarely curative and often limited^42,43^. Hence, in the present study, we aimed to develop a novel class of rabbit-derived VL sdAb-based ADC for the treatment of cNHL, that serves as an animal model for hNHL. To achieve this, a highly diverse immune library of rabbit VL sdAbs against primary canine NHL cells was successfully constructed to ensure the presence of antibodies against different targets in the disease setting. Due to their unique B-cell ontogeny, rabbit antibody-derived libraries present a highly distinctive and diverse antibody repertoire, rich in in vivo pruned binders of high diversity, specificity and affinity^33^. Importantly, rabbits are evolutionarily distant from mice and rats, so epitopes that are not immunogenic in rodents can be recognized by rabbit mAbs, increasing the targetable epitopes and facilitating the generation of mAbs that cross react with other species—a key aspect for clinical translation. Our data showed that rabbit immunizations with intact B-cell canine non-Hodgkin lymphoma primary cells resulted in a specific and selective high-tittered antiserum against cNHL epitopes. The strong and specific response generated allowed the construction of an antibody library highly diverse and representative (3.4 × 10^8^). Thereafter, a strategy of an in vitro whole-cell phage display, followed by an in vivo phage display in a cNHL xenograft murine model, was used to select the best sdAbs targeting antigens in their natural conformation and tumor microenvironment. This methodology allowed the selection of both VL-phages that bind and internalize to the tumor cells. One of the main benefits of this in vivo selection is its innate negative selection feature. This enables the reduction of the off-target tissue and protein interactions by eliminating non-specific ligands, enriching the recovery of specific ligands that specifically target the tumor. Despite its advantages, in vivo phage display selection remains relatively unexplored^44,45^. To date, few studies on cancer models have been reported. Soendergaard et al. used in vivo phage display selection to identify an ovarian cancer targeting peptide^46^. Another study of Veleva et al., applied a combination of in vitro and in vivo screening to isolate a peptide that is selective for circulating bone marrow derived cells from angiogenic Lung Lewis carcinoma tumors^47^. To the best of our knowledge, this is the first time that an in vivo selection has been applied in the selection of sdAbs against lymphoma and for ADC development. Overall, the obtained results reinforce in vivo phage display as a powerful technology that has the potential to expand the repertoire of targetable tumor receptors, while simultaneously confirms the availability of the in vivo epitopes and generates highly specific antibodies for tumor targeting. After obtaining a specific pool of sdAbs for cNHL targets by phage display selection, we selected the best VL sdAb candidates based on their binding activity to cNHL targets and expression via ELISA screening. This study was complemented with a sanger and NGS analysis of the recovered in vivo biopanning with the initial library. By investigating the prevalence of each sequence in each sample, NGS analysis revealed the specific enrichment attained by the phage display selection and reinforced the ELISA results proposing the selection of C5 VL-sdAb as the most promising clone. To confirm C5 suitability as our ADC targeting moiety, its binding and cellular internalization on cNHL cells was further characterized by flow cytometry and immunofluorescence. Overall, these results proved the specificity of the interaction between the sdAb and cNHL cells and the subsequent complex internalization, which are key attributes for the success of an ADC. Importantly, the biodistribution studies demonstrated that C5 exhibited a good tumor uptake with an accumulation percentage of around 1.5% ID/g after 15 min of injection, meaning that an adequate amount of our sdAb is reaching the tumor. The biodistribution profiles of different antibody formats were already compared by different groups. Schneider et al.^48^, compared different antibody formats, including scFvs, and the percentages of tumor uptake obtained were between 1 and 1.5% for 25 min and 3 h after injection. Another study conducted by Duray et al.^49^ using sdAbs, revealed a tumor uptake of 2.22% and 0.79% for 48 h post-injection. The values obtained for small fragments are in the same range as those we obtained for our sdAb, C5, leading us to conclude that our data resulted in a good tumor uptake. Moreover, C5 biodistribution profile presents a rapid clearance from the major organs, except for the organs related with the excretory paths (liver and kidney) and in the spleen. This fast clearance from the systemic circulation is mostly advantageous given that it results in shorter effective circulation times, mitigating the possibility of off-target release. Finally, and importantly to proceed with ADC development, the presence of the free Cys80 at the surface of C5 was confirmed by modelling its tridimensional structure using a protein structure prediction software.

Once the target-specific antibody is selected, the choice of a potent payload becomes critical. To design our ADC, we explored the potent, well-characterized and clinically validated payload – SN-38. SN-38 acts as a Topo I inhibitor through binding and stabilization of the Topo I-DNA cleavage complexes, leading to accumulation of DNA damage and apoptosis^50,51^. Cancer cells express high yields of Topo I, making its expression 14–16 times higher than in normal cells. The increase of this enzyme yield is particularly observed in certain types of cancer, including NHL. Additionally, a recent study reported its use in the construction of a sdAb-based ADC which suggests it possesses adequate physicochemical properties (hydrophobicity and potency) to build our new sdAb-drug conjugate^52^. Moreover, SN-38 has already proven clinical efficacy, being used as payload in a recently FDA-approved ADC, Sacituzumab govitecan (Trodelvy), indicated for the treatment of metastatic triple negative breast cancer^53^. Therefore, considering its high activity, we envisioned the construction of an ADC featuring both C5 and SN-38, connected by a linker. The linker is another key component of the ADC as it governs the stability of the conjugate and avoids random release of the cytotoxic payload in circulation. In the new generation of ADCs, the linker evolved from a stable and inert spacer into a functional structure capable of responding to specific stimulus and releasing the payload selectively at the target tissues. This selectivity is achieved by taking advantage of the exquisite properties of tumoral microenvironment, which includes acidic pH, high concentration of glutathione and overexpression of proteolytic enzymes. In addition, high concentrations of reactive oxygen species (ROS) is also one of the hallmarks of cancer^54^. Recently, we reported the development of a new bioconjugation linker (diazaborines) that is susceptible to oxidation in the presence of ROS^55^ and is an excellent option for the preparation of a tumor-targeted ADC. DAB-SN-38, a highly complex molecule featuring the SN-38 drug, a ROS-responsive diazaborine linker and a maleimide bioconjugation handle, was conjugated to C5 at the free Cys80 to generate the stable and homogenous C5-DAB-SN-38, with a drug-to-antibody ratio (DAR) 1. The expected DAR was confirmed by reverse phase-HPLC which displayed > 95% of single modified sdAb. The obtained data showed that C5-DAB-SN-38 promoted cell death on the canine lymphoma cell line, CLBL-1. In addition, our results demonstrated that C5-DAB-SN-38 cytotoxicity is associated with DNA-Topo I inhibition, as expected. Noteworthy, these results indicate that the ADC C5-DAB-SN-38 was stable generated and presented a high cytotoxic activity against canine diffuse large B-cell lymphoma under the nM range, revealing the potential of these rabbit-derived sdAbs as ADC moieties.

In conclusion, the results presented herein validate a highly efficient approach for the discovery and generation of anti-tumor antibodies for ADC development. The combination of an immunized library using NHL cells from canine patients with an innovative in vivo phage display on a cNHL xenograft murine model enabled the selection of highly specific antibodies against NHL. Importantly, by exploring the promising properties and unique characteristics of rabbit derived VL sdAbs scaffolds we were able to engineer a potent ADC with a DAR 1 by conjugating the SN-38 payload into the free exposed Cys80 of the VL framework. Thus, this work creates a new research avenue in the development of ADCs for cancer treatment by urging the emergence of a novel class of rabbit-derived sdAb-based products with versatile properties for payload conjugation. In addition, the opportunity to carry clinical studies in pet dogs with naturally occurring canine B-cell lymphoma may also provide resources with realistic perspectives for clinical translation in the immune-oncology field.

## Materials and methods

### Cell lines and culture

CLBL-1, Jurkat and HEK293T cell lines were obtained and maintained as previously described^25,35,36,39,56^.

### Rabbit immunization and antibody library construction

All animal-handling procedures were performed according to EU recommendations for good practices and animal welfare and approved by the Animal Care and Ethical Committee of the Veterinary Medicine Faculty (Protocol_0050132016). All methods were performed in accordance with the relevant guidelines and regulations. The study was conducted according ARRIVE guidelines. One female New Zealand White rabbit (Charles River) was immunized with lymph node primary cells derived from a canine multicentric lymphoma biobank (cNHL) previously established and characterized by our group^34^. To induce a strong and specific immune response against NHL receptors, rabbit was immunized and boosted with 1 × 10^7^ of cNHL primary cells isolated from tumor affected lymph node from two patients (ID5 and ID6)^34^ diagnosed with Diffuse Large B-Cell Lymphoma (DLBCL). For that purpose, tumor cells isolated from lymphoma-affected lymph nodes were thawed, washed in PBS and after confirmation of cell viability, resuspended in 1 ml of PBS. The injections were administered subcutaneously at 2–3 weeks intervals for 3 months. Before each immunization, blood was harvested from the marginal ear vein for serum isolation. Five days after the final boost, rabbit was sacrificed by cardiac puncture exsanguination, following propofol anesthesia, and spleen and bone marrow were harvested for total RNA isolation, cDNA synthesis and VL immune library was constructed as previously described^24,25,29^.

### Characterization of rabbit immune response

The rabbit immune response was monitored by cell ELISA sera testing using the cNHL primary cells (ID5 and ID6)^25,34^ used in the immunizations and CLBL-1 cells, a canine DLBCL stable cell line^35,36^, as described previously^25^. Pre-bleed sera was used as control. Each serum was also analyzed for its binding properties against cNHL cells by flow cytometry. For that, ID5 and ID6 cNHL cells and CLBL-1 cells were prepared. Cells were washed twice in 0.5% PBS-BSA and incubated with the rabbit pre-bleed and final bleed (1/3000) for 30 min at 4 °C. Cells were then washed with cold 0.5% PBS-BSA three times and incubated with secondary antibody (Alexa Fluor-647 Goat Anti-Rabbit IgG) at 1/10,000 in 0.5% PBS-BSA for 30 min at 4 °C. Cells were washed with 0.5% PBS-BSA three times and submitted to flow cytometry analysis (FACSCalibur). Unstained cells were used as negative control for voltage settings. For multiple-color sorts, single color controls were used for compensation settings. Data was analyzed by FlowJo software version 10 (FlowJo LLC).

### Phage display selection of VL sdAbs targeting cNHL

The phage library displaying VL sdAbs was first panned using a subtractive cell phage display protocol as previously described by Carlos Barbas^37,38^ and our studies^25^, and that included a negative selection on HEK293T cells followed by a positive selection on CLBL-1 cells. Then, after three rounds of in vitro selections, an additional panning was performed in vivo in a xenograft CLBL-1 murine model^39^. All animal-handling procedures were performed according to EU recommendations for good practices and animal welfare and approved by the Animal Care and Ethical Committee of the Veterinary Medicine Faculty (Protocol_0050132016). All methods were performed in accordance with the relevant guidelines and regulations. The study was conducted according ARRIVE guidelines. Briefly, female 6–8-wk-old SOPF/SHO SCID mice (Charles River) were maintained as mentioned before^39^. Then, the tumors were induced as established previously^39^. When tumors reached a minimum volume of 100 mm^3^, three SCID mice were intravenously injected into the tail vein with 100 µl of phage (1 × 10^10^ pfu/ml) freshly prepared from the third in vitro selection round. Phages were allowed to circulate for 60 min, then mice were sacrificed, perfused and xenograft tumors were removed and weighted. Following tumor homogenization in 70 µm cell strainers (VWR, Radnor, PA, USA) phages were recovered by incubating the homogenized tumor with trypsin, as described previously^24^. To elute the internalizing phages, the cell pellet obtained after the trypsin elution was washed 3 × with PBS and centrifuged at 10,000 × g at 4 $^\circ$C, 5 min. Then, the cell pellet was resuspended with 200 µl of 0.1 M triethylamine, incubated 10 min and neutralized with 50 µl of 1 M Tris 7.5. The eluted phages were treated as described before^24^.

### Analysis of phage display enrichment by next generation sequencing and VL sdAb lead selection by ELISA

To analyse the enrichment and profile obtained after the in vivo phage display selection, we performed a next generation sequencing (NGS) analysis as described previously^24^. As control the initial immune VL sdAb library was sequenced and the data obtained compared with the selected library. To select the most promising VL sdAbs with ADC properties, phagemid DNA from the in vivo output selection (internalizers output) was cloned into the PT7-PL (PT7-peptide leader) vector, transformed into Escherichia coli BL21 (Lucigen) and autoinduced as previously reported^24,25^. To evaluate the relative binding activity and specificity an ELISA was performed as previously mentioned^25^. The best lead candidates in terms of binding and expression levels, were selected and sequenced at Eurofins company. Sequence analysis was performed as mentioned before^24,57^.

### Production and purification of VL sdAbs

The three best VL sdAbs were selected according to cNHL cell binding activity and expression yields. C5 was chosen as the best lead candidate to be expressed and purified. For that, C5 was cloned into the pET21 expression vector (Sigma-Aldrich, St. Louis, MO, USA) and transformed in E. coli BL21 (Lucigen). C5 was expressed as described previously^24^. After expression, bacteria were harvested by centrifugation (1500xg, 15 min, 4 °C), and resuspended in 50 ml of initial buffer (50 mM HEPES, 1 M NaCl, 10 mM Imidazole, 2 M Urea, 5 mM CaCl2, 1 mM β-mercaptoethanol, and pH = 8) supplemented with protease inhibitors (Roche). Cells were lysed by sonication and the inclusion bodies were recovered by centrifugation (7500xg, 30 min, 4 °C) as described previously^26^. Refolding was performed by step wise dialysis, according to Gouveia et al., 2017^26^. After that, C5 was purified by size exclusion chromatography (SEC) as previously mentioned^24^.

### Immunofluorescence microscopy and flow cytometry of C5

1.5 × 10^5^ of CLBL-1 or Jurkat cells were plated on ibidi µ-Slide 8 Well Glass Bottom (Ibidi, Fitchburg, WI, USA) and incubated for 24 h at 37 °C in a humidified atmosphere of 5% CO2. Then, 3 µM of C5 was added to the cells and incubated for 90 min at 37 °C. After incubation, cells were washed twice with PBS, fixed with 4% PFA for 15 min at room temperature, permeabilized with 0.1% Triton X-100 for 10 min at RT, washed, blocked with 0.1% Triton X-100 and 3% PBS/ BSA and incubated overnight with rat anti-HA (Roche, 1/50) at 4 °C. Next day, cells were washed twice with PBS and incubated with anti-rat Alexa Fluor-488 (1/500) for 1 h at RT. After washing, DAPI Vectashield (Vector Labs, Burlingame, CA, USA) was added to the cells. Image acquisition was performed on a confocal point-scanning Zeiss LSM 880 microscope (Carl Zeiss, Germany) equipped with a Plan-Apochromat DIC X63 oil objective (1.40 numerical aperture). Diode 405–30 laser was used to excite DAPI, and argon laser in the 488-nm line to excite Alexa Fluor-488. In the Airyscan acquisition mode, × 1.80 zoom images were recorded at 1024 × 1024 resolution. ZEN software was used for image acquisition and Fiji software was used for image processing. For Flow Cytometry analysis, 1 × 10^6^ of CLBL-1 or Jurkat cells were treated as described previously^25^. C5 was incubated for 90 min at 37 °C.

### Biodistribution studies and tumor targeting

To evaluate the biodistribution and tumor targeting in a xenograft CLBL-1 murine model^39^, C5 was radiolabeled with the radioactive precursor [^99m^Tc(CO)3(H_2_O)3] + prepared from an IsoLink kit (Covidien, Ireland). Radiochemical purity (RP) was checked by Reversed-phase high-performance liquid chromatography (RP-HPLC) and instant thin-layer chromatography silica gel (ITLC-SG, Agilent Technologies, USA). In brief, fac-[^99m^Tc(CO)3(H_2_O)3] + solution was added to a nitrogen-purged closed glass vial containing a solution of His-tag with C5 to obtain a final concentration of 1 mg/ml. The mixture was incubated for 45–60 min at 37 ºC and then a ITLC-SG analysis using 5% HCL (6 M) solution in MeOH as eluent was performed to evaluate the RP of ^99m^Tc(CO)3-C5. While [^99m^Tc(CO)3(H_2_O)3] + and [^99m^TcO4]- migrate in the front of the solvent (Rf = 1), the ^99m^Tc(CO)3-C5 remains at origin (Rf = 0). Radioactivity distribution on the ITLC-SG strip was evaluated using a miniGita Star scanning device (Elysia-Raytest, Germany) coupled with a Gamma BGO-V-Detector (Elysia Raytest). For purification and concentration of the ^99m^Tc-labeled sdAb, a 3K Amicon (Merck Milipore) was used. ^99m^Tc(CO)3-C5 diluted in PBS was used for the biodistribution studies after RP determination by ITLC-SG. For that, mice were intravenously injected in the tail vein with 100 µl of ^99m^Tc(CO)3-C5 and sacrificed by cervical dislocation at 15 min, and 3 h after injection. Radioactivity was measured using a dose calibrator (Carpintec CRC-15W). After the removal of the tumor and tissues of interest, their radioactivity was measured using a γ-counter (Berthold, Germany). The uptake was represented as a percentage of injected activity dose per gram of organ or tissue (%ID/g). To confirm the results and C5 tumor uptake, western blot analysis was performed as described previously^24^. All animal-handling procedures were performed according to EU recommendations for good practices and animal welfare and approved by the Animal Care and Ethical Committee of the Veterinary Medicine Faculty (Protocol_0050132016). All methods were performed in accordance with the relevant guidelines and regulations. The study was conducted according ARRIVE guidelines.

### 3D VL sdAb structure prediction

The tridimensional structure of C5 was predicted using the Phyre2 protein fold recognition server (http://www.sbg.bio.ic.ac.uk/phyre2)^58^. The best model was obtained using the structure of an immunoglobulin light chain as a template (PDB entry: 1BJM) with a level of confidence of 100% and a coverage of 98%. Visual representations of the obtained model were produced using the UCSF Chimera software^59^.

### Synthesis and characterization of the Linker-Payload (DAB-SN-38)

The synthesis of DAB-SN-38 was performed as previously reported^55^.

### Bioconjugation of C5 with SN-38

To conjugate the C5 with SN-38, a PBS pH 7.4 solution containing C5 (10 µM) and TCEP (1.5 equiv., 3.5 mM), DAB (20 equiv., 9 mM, DMSO) was added, and the solution was mixed during 90 min at 25 $^\circ$C. The expected conjugate was evaluated after 90 min by High-Resolution Mass Spectrometry, recorded in a Thermo Scientific Q Exactive Plus (Thermo Scientific). The final immunoconjugate C5-DAB-SN-38 was detected and purified by dialysis using a Pur–A–Lyzer™ Midi Dialysis Kit with a 3.5 kDa cut-off. The mass spectra were deconvoluted using MagTran software.

### Cytotoxic assay

To determine the effect of C5-DAB-SN-38 on CLBL-1 and Jurkat cell proliferation, a cell viability assay was performed as described previously^56^. Briefly, cells were seeded at a density of 6 × 10^4^ well in 200 µl of culture medium and subjected to increasing concentration (2.5 μM to 12.5 nM) of each compound (C5, C5-DAB-SN-38 and SN-38). After 48 h treatment, cell viability was assessed using WST-1, following the manufacturer’s instructions. Absorbance at 450 nm was measured using the iMark microplate Reader (Bio-Rad). Two replicate wells were used to determine each data point and three independent experiments were carried out in different days. Best-fit EC50 values of each formulation were calculated using GraphPad Prism software (version 9.2.0, San Diego, CA, USA) using the log (inhibitor) vs response (variable slope) function.

### DNA-Topo I activity assay

Topo I activity on C5-DAB-SN-38 was determined using the Human Topoisomerase I Assay Kit (Topogen, Buena Vista, CO, USA) according to the manufacturer’s instructions. Samples were loaded on a 1% agarose gel and ran in 1 × TAE buffer. Gel was stained with ethidium bromide for 45 min and destained in distilled water. Relaxed DNA, SN-38 and C5 were used as controls.

## Supplementary Information

Supplementary Information.

## Data Availability

All data generated or analyzed during this study are included in this published article and its supplementary information files.
