# Supplementary material for: Rabbit derived VL single-domains as promising scaffolds to generate antibody–drug conjugates
Source: Sci Rep. 2023 Mar 24;13:4837. doi: 10.1038/s41598-023-31568-x (PMC10038998; doi:10.1038/s41598-023-31568-x)
Supplement: Supplementary file 1 — Supplementary Information. [file 41598_2023_31568_MOESM1_ESM.docx]

**
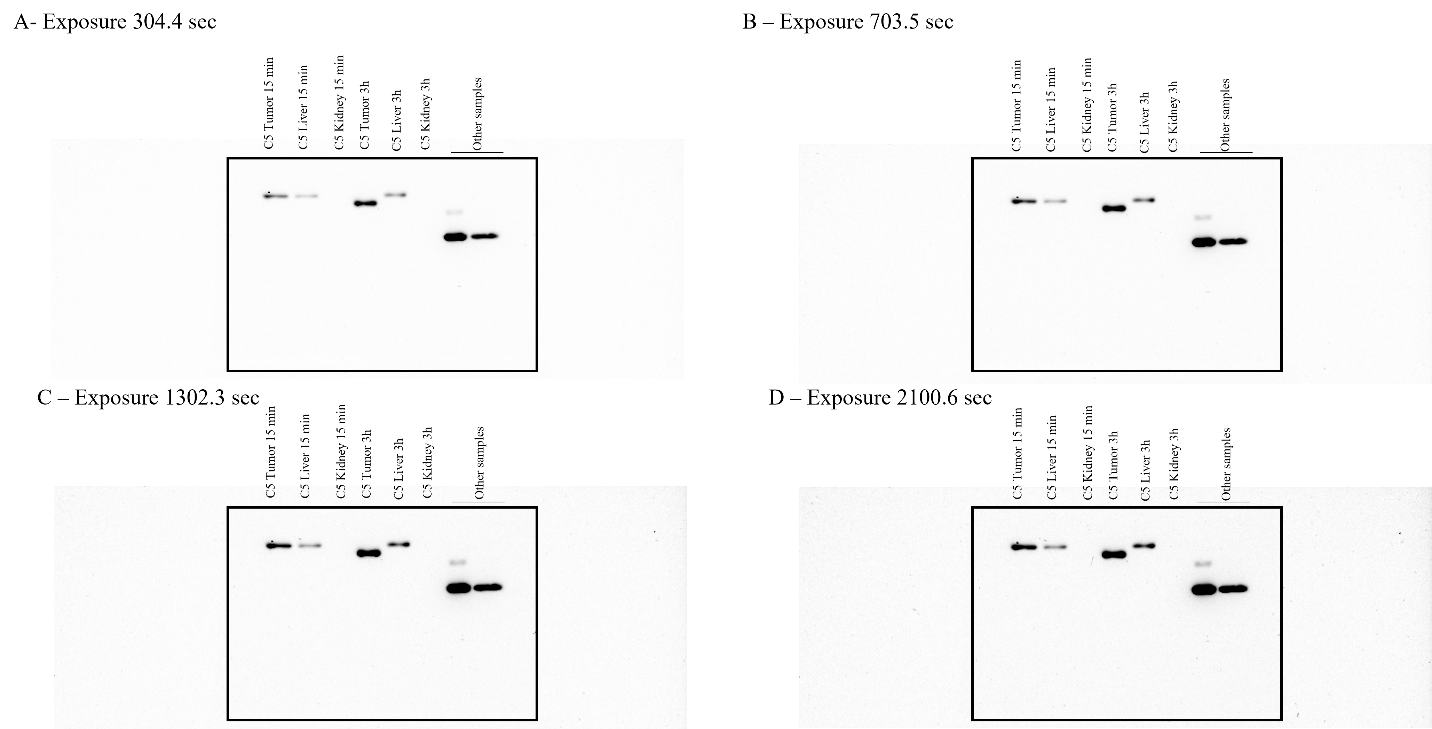
**

**Supplementary Figure 1:** **Different exposures of the original blot of Figure 5C.** All revelations were for sdAb C5 shown in Figure 5C. Figures A and B are for low exposures. C and D for high exposures. D was the exposure shown in Figure 5C. Black line indicates the borders of the blot. Protein detection was performed by chemiluminescence using Luminata ForteWestern HRP and acquired using the ChemiDoc XRS+ imaging system (Bio-Rad).


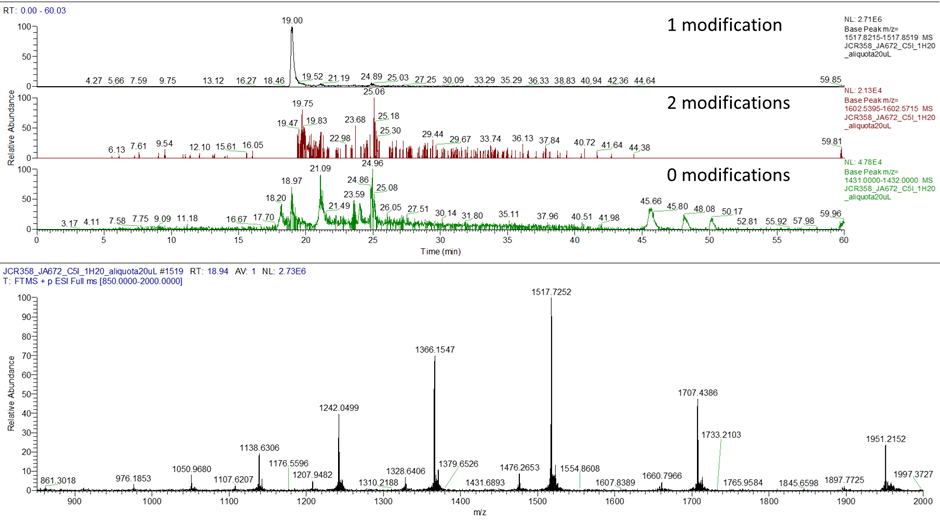


**Supplementary Figure 2:** **Evaluation of the DAR of C5-DAB-SN-38 by HPLC-MS**. Extracted Ion Current chromatogram of: (Top) Single-modified, (Middle) Double-modified and (Bottom) unmodified C5. EIC of the single modification displays an intensity of 2.71 × 10^6^, compared to 10^4^ intensities of double-modified and unmodified C5. These numbers suggest >95% of single-modified C5, validating the presence of a homogenous conjugate.


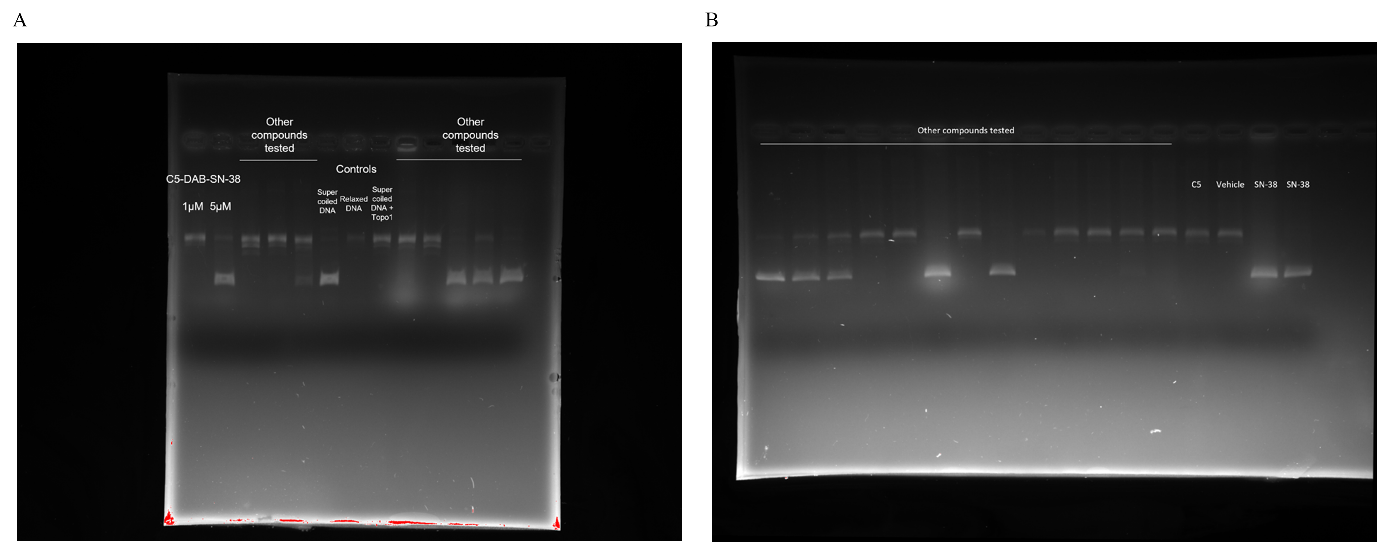


**Supplementary Figure 3:** **Original gel of Figure 7D.** A) Original gel with samples C5-DAB-SN-38 (1μM and 5μM) and Controls (Super coiled DNA, Relaxed DNA and Super coiled DNA + Topo1). B) Original gel with samples C5, Vehicle and SN-38.
